# Supplementary material for: Molecular Targets Implicated in the Antiparasitic and Anti-Inflammatory Activity of the Phytochemical Curcumin in Trichomoniasis
Source: Molecules. 2020 Nov 14;25(22):5321. doi: 10.3390/molecules25225321 (PMC7697451; doi:10.3390/molecules25225321)
Supplement: Supplementary file 1 [file molecules-25-05321-s001.pdf]

Supplementary Material

Evaluation of cell viability and cytotoxicity of curcumin in RAW 264.7 cells by tetrazolium (MTT) assay

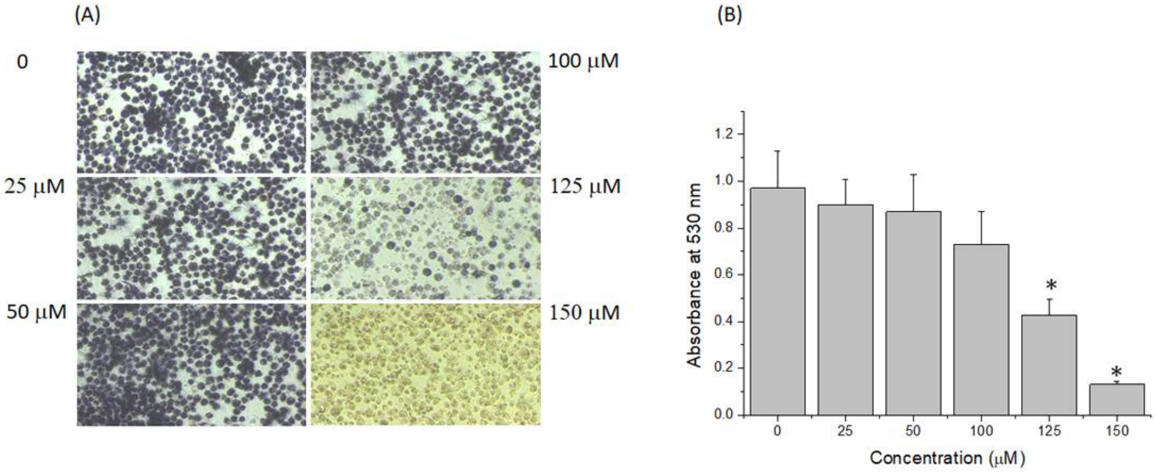

Figure S1. (A) Microphotographs of RAW 264.7 cells stained with MTT and incubated for 24 hours with curcumin at concentrations between 25–150 μM. (B) Quantification of formazan levels produced by RAW 264.7 cells incubated for 24 h with curcumin at concentrations between 25–150 μM. In the graph, each bar represents the mean ± standard deviation (n = 3) of the absorbance values at 530 nm generated by 10^6 cells / mL using the MTT assay following the protocol previously described by *Sueiro et al.* (2020). Asterisks indicate statistically significant differences ($P < 0.01$) compared to the control group to which no curcumin has been added.

Reference:

Sueiro-Benavides RA, Leiro-Vidal JM, Salas-Sánchez AÁ, Rodríguez-González JA, Ares-Pena FJ, López-Martín ME. Radiofrequency at 2.45 GHz increases toxicity, pro-inflammatory and pre-apoptotic activity caused by black carbon in the RAW 264.7 macrophage cell line. *Sci. Total Environ.*, **2020**, 4, 142681. doi: 10.1016/j.scitotenv.2020.142681. Epub ahead of print. PMID: 33071139.
